# Limited alcohol consumption and lower risk of diabetes: can we believe our own eyes?

**DOI:** 10.1093/ajcn/nqac258

**Published:** 2022-10-17

**Authors:** Kenneth J Mukamal, Joline W J Beulens

**Affiliations:** Department of Medicine, Beth Israel Deaconess Medical Center, Boston, MA, USA; Department of Epidemiology & Data Science, Amsterdam UMC Medical Center, location Vrije Universiteit, Amsterdam, Netherlands; Amsterdam Cardiovascular Sciences, Amsterdam Public Health, Amsterdam, Netherlands; Julius Center for Health Sciences and Primary Care, University Medical Center Utrecht, Utrecht, Netherlands

See corresponding article on page 1507.

Although excessive alcohol consumption can cause chronic pancreatitis (1) and eventually exocrine and endocrine pancreatic insufficiency (2), the effect of alcohol consumed within recommended limits (i.e., ≤2 drinks/d in men, ≤1 drink/d in nonpregnant women) on the risk of diabetes remains a long-standing and ongoing source of controversy (3). Although the elegant analyses conducted by Ma et al. (4) in this issue of *The American Journal of Clinical Nutrition* will not bring this controversy to a conclusion, they illustrate, in part, why it has sometimes felt, to paraphrase Chico Marx in *Duck Soup*, so difficult to believe our own eyes.

At heart, this debate resembles comparable ones for cardiovascular disease, gallstone disease, and other conditions that appear to be less common among limited drinkers than among abstainers in observational cohort studies. This is certainly true for diabetes, where meta-analyses of prospective cohort studies have confirmed the association of limited alcohol consumption with lower risk (5). However, skeptics have repeatedly cautioned that this evidence may not be believable (6), largely because of residual confounding and so-called abstainer bias (which simply represents confounding by subclinical illness) stemming from the reduced alcohol consumption that accompanies age, lower socioeconomic status, frailty, and chronic disease.

To overcome these methodological issues, many investigators have turned to genetic instrumental variable analyses or, speciously, “Mendelian randomization (MR).” This approach tests the associations with specified endpoints of genetic variants (i.e., proxies) that are themselves associated with alcohol consumption (or any other exposure of interest); if several assumptions are met, it is possible to estimate the association of alcohol consumption with these endpoints from the association of its proxies with those same endpoints.

With the widespread availability of genome-wide association studies, investigators have taken to conducting genetic instrumental variable analyses for essentially every exposure, whether or not the necessary assumptions [reviewed elsewhere (7)] are met. This is certainly true for alcohol, which has been the subject of dozens of these analyses, all of which have relied upon variants in the genes encoding alcohol-metabolizing enzymes (sometimes in conjunction with other, weaker variants) (3). Unfortunately, these studies have demonstrated variable degrees of rigor (8) and have relied upon variants that are proxies for alcohol use disorder, not for limited alcohol consumption (9). It may not be surprising, therefore, that this approach has failed to confirm a lower risk of diabetes with alcohol consumption and, in some cases, has suggested higher risk (8).

The case of alcohol and diabetes is unique, however, in that randomized trials of alcohol consumption among nondiabetic individuals have already tested its association with central aspects of diabetes pathophysiology. No equivalent to insulin resistance or glycemia itself exists for cardiovascular disease, for example, precluding this approach for other diseases, but this apposition enables a direct comparison of standard epidemiology (i.e., our own eyes), genetic epidemiology, and truly gold-standard evidence in this unique instance. What have these trials demonstrated?

In brief, they firmly support the results of conventional epidemiology. Our meta-analysis of 14 such trials showed that limited alcohol consumption significantly reduced fasting insulin and glycated hemoglobin concentration (10). Although these trials were generally short-term, every existing trial among adults with diabetes that has spanned months to years has also demonstrated significant improvements in insulin resistance and/or glycemia (11–13).

How should we reconcile results from actual randomized trials with genetic studies that suggest otherwise? Ma et al. provide several important clues to this discrepancy. The authors studied >300,000 current drinkers in the UK Biobank, who were followed for ∼11 y for the occurrence of type 2 diabetes. The authors excluded nondrinkers, fully eliminating concerns about abstainers, and adjusted for key confounders including socioeconomic status. Even so, alcohol consumption was nonlinearly associated with risk of diabetes, with the lowest risk among consumers of between 100 and 200 g (i.e., ∼8–16 drinks) per week. Moreover, the authors observed a statistically significant 14% lower risk of type 2 diabetes for those consuming alcohol with meals than for those drinking outside meals; individuals with varying patterns showed intermediate risk. This finding persisted even with adjustment for actual drinking amount, although individuals who reported consuming alcohol only with meals were more likely to be high-frequency, low-quantity consumers. Of note, that very pattern has previously been associated with lower risk of diabetes (14). Thus, limited alcohol consumption was associated with lowest risk of diabetes, but specifically when consumed in a pattern that minimizes upward excursions in blood alcohol concentrations (as drinking with meals and low-quantity intake both do).

Unfortunately, genetic variants are blunt instruments that are ill-suited for the very complexity that alcohol intake demonstrates. Whether genetic instrumental variable analysis can ever identify known nonlinear relations is unproven, yet Ma et al. again demonstrate that dose matters for alcohol. No evidence exists that instruments specific for a low-risk drinking pattern can be derived, yet the current report also demonstrates that drinking patterns that include quantity, frequency, and consumption with meals must be taken into account. Of note, trials of alcohol on metabolic endpoints have essentially always provided alcoholic beverages with meals, in limited quantities per drinking day, and on a (almost) daily basis—exactly the pattern that Ma et al. confirmed to be associated with lower risk of diabetes in the UK Biobank.

Although these findings illustrate exactly why complexity in multidimensional exposures like alcohol must be retained, they are also limited to the case of alcohol consumption and diabetes. As others noted years ago (15), only a large-scale randomized trial can definitively determine the full range of health effects of limited alcohol consumption beyond glucose metabolism. No amount of standard or genetic epidemiology can replace a definitive trial, and it behooves the ASN and similar bodies to call for one and overcome the inertia and nonscientific forces that stand in the way (16). To conclude with a paraphrase of Groucho Marx, as we consider the need for a randomized trial to produce better evidence on alcohol, “OK, it's time.”

## Notes

The authors reported no funding received for this study.
